# Enhancing the Activity of Ribokinase from *Thermus* sp. 2.9 Through Rational Design for the Cascade Synthesis of Modified Nucleotides and Nucleosides

**DOI:** 10.3390/ijms27198911

**Published:** 2026-10-07

**Authors:** Evgeniy A. Zayats, Yulia A. Abramchik, Maria A. Kostromina, Ilya V. Fateev, Andrey A. Karanov, Alexandra R. Sharafutdinova, Vasiliy A. Ptushkin, Irina D. Konstantinova, Andrey V. Golovin, Roman S. Esipov

**Affiliations:** 1 Shemyakin–Ovchinnikov Institute of Bioorganic Chemistry, Russian Academy of Sciences, Moscow 117997, Russia; 2 Faculty of Bioengineering and Bioinformatics, Lomonosov Moscow State University, Moscow 119991, Russia

**Keywords:** cascade synthesis, enzyme design, boltz-2, ribokinase, modified nucleosides, modified nucleotides

## Abstract

The pursuit of efficient approaches for the production of promising antiviral and cytostatic pharmaceuticals is a crucial direction in medical biotechnology, especially after the COVID-19 pandemic. Modified nucleosides and nucleotides represent a highly promising class of chemicals for the development of drugs against cancer and viral infections. Being an alternative to traditional chemical synthesis, cascades of nucleic exchange enzymes offer single-step synthesis of such compounds with exceptional stereo- and regioselectivity. Furthermore, employing thermostable enzymes for such cascades offers the advantages of increased substrate solubility, lower solution viscosity, prevention of contamination, and simplified biocatalyst purification via thermal denaturation. The first biocatalyst for two distinct cascade pathways of nucleotide and nucleoside synthesis is ribokinase. In this article, we report improving the activity of ribokinase from *Thermus* sp. 2.9 (*Tsp*RK) for the cascade synthesis of modified nucleosides and nucleotides. We have employed a rational design approach, including Boltz-2 modeling and sequence conservation analysis. We have engineered two mutants, T244I and N272H, achieving a 3-fold increase in ribose phosphorylation rate (*k_cat_*). Improving the enzymatic activity of a thermostable ribokinase will serve as the basis for further optimization of cascade synthesis of pharmaceutical modified nucleosides and nucleotides.

## 1. Introduction

One of the most important challenges for modern biotechnology is the development of efficient technologies for the production of drugs: both broadly applied pharmaceuticals and promising experimental compounds [1,2]. This research direction is especially relevant for antiviral drugs, highlighted by the recent COVID-19 pandemic [3,4,5]. The rapid rise and spread of SARS-CoV-2 infections highlights that vaccines are not sufficient for disease management. Development of safe and efficient treatments against viral infections necessitates the search for new biologically active compounds [6,7]. Moreover, cancer treatment also remains a highly relevant healthcare challenge, requiring the development of new cytostatic drugs [8,9].

Nucleobases, nucleosides, and nucleotides represent a highly promising class of chemicals. Modified analogs of these compounds can interfere with nucleic acid metabolism, thus displaying broad activity against viruses and tumors [10,11,12,13,14,15]. Another area where the modification of nucleobase and pentose moieties is highly relevant is the development of oligonucleotide therapeutics, such as mRNA vaccines, antisense oligonucleotides, small interfering RNAs, and aptamers with reduced immunogenicity, increased affinity and stability [16,17].

However, the classical chemical synthesis of nucleosides and nucleotides necessitates protection and deprotection of chemical groups, along with separation of stereo- and regioisomers [18,19,20,21,22]. As a promising alternative to such approaches, one can employ multienzymatic cascades, which offer single-vessel synthesis of desired modified compounds without the challenges of stereo- and regioisomerism [19,23]. Nevertheless, biocatalytic approaches have their limitations, including limited activity of some enzymes or their narrow substrate specificity. However, the limitations of natural enzymes can be overcome through rational design approaches and directed evolution [24,25,26,27,28,29].

**Figure 1:**
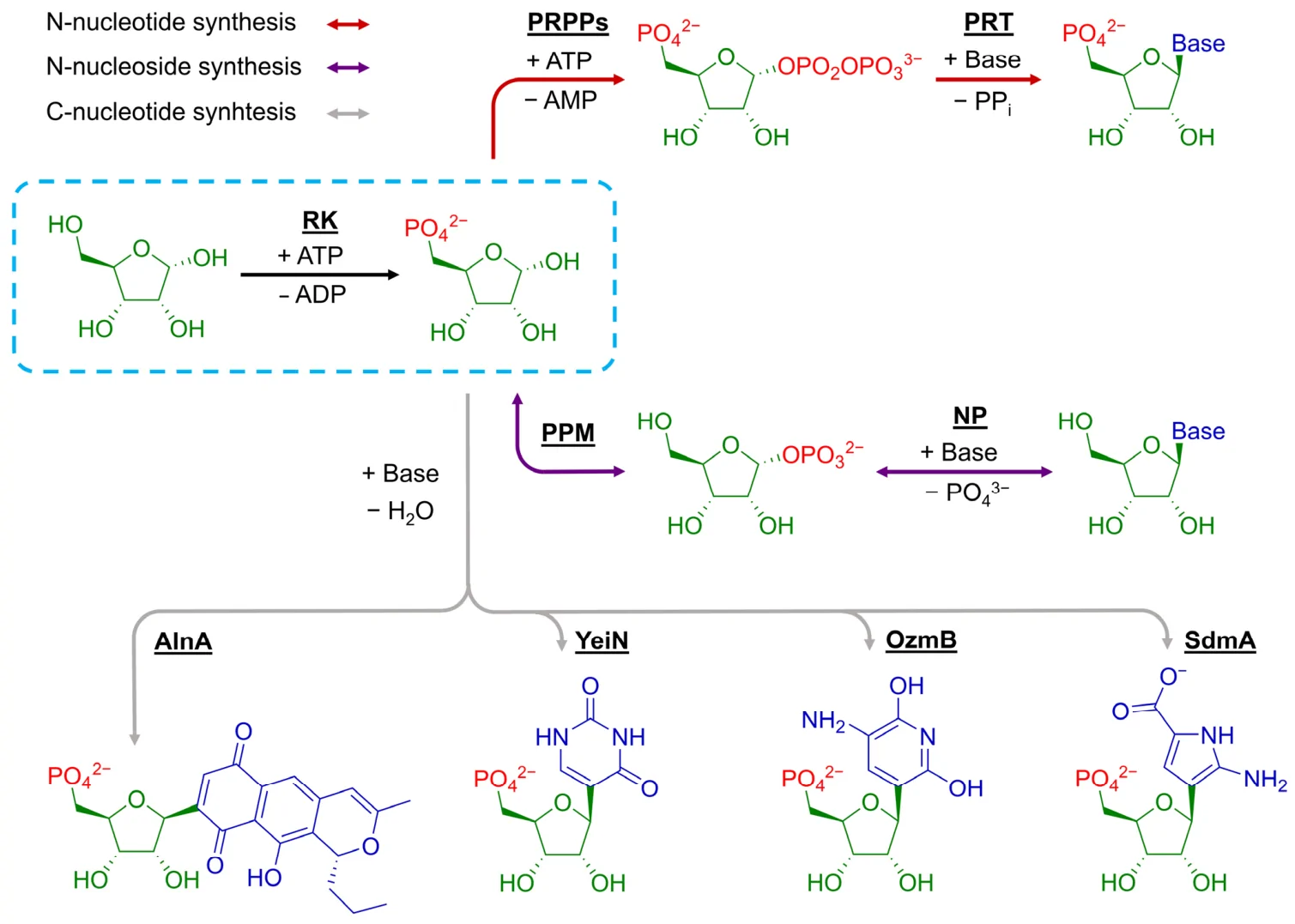
Applications of ribokinase for the enzymatic synthesis of nucleosides and nucleotides. Base—nitrogenous base, PP_i_—inorganic pyrophosphate. Carbohydrate and nucleobase moieties are highlighted in green and blue, respectively, while phosphate and pyrophosphate groups are highlighted in red.

Previously, we have developed cascades of nucleic exchange enzymes from thermophilic and mesophilic organisms for the synthesis of modified nucleosides and nucleotides [30,31]. The scheme of these cascades is shown in Figure 1. All of these cascades begin with commodity reagents such as D-ribose, 2-deoxy-D-ribose or D-arabinose. These pentofurranoses are introduced into the cascade as carbohydrate moiety donors via the first enzyme—ribokinase (RK, EC: 2.7.1.15). This enzyme phosphorylates the pentose sugars through the 5′-hydroxyl group, using ATP as the second substrate [32,33,34,35,36,37].

From the resulting product ribose-5-phosphate, the synthetic pathway splits into two different cascades. In the case of nucleotide synthesis, phosphoribosylpyrophosphate synthetase (PRPPs, EC 2.7.6.1) transfers the pyrophosphate group from ATP to ribose-5-phosphate [31,38,39]. The resulting phosphoribosylpyrophosphate (PRPP) is used as the carbohydrate donor in the final reaction of the cascade—nucleotide synthesis using phosphoribosyltransferases (PRT): adenine phosphoribosyltransferase (APRT, EC 2.4.2.7), hypoxanthine-guanine phosphoribosyltransferase (HGPRT, EC 2.4.2.8) or nicotinamide phosphoribosyltransferase (NAMPRT, EC 2.4.2.12) [31,38,39].

Alternatively, ribose-5-phosphate can be isomerized into ribose-1-phosphate by phosphopentomutase (PPM, EC 5.4.2.7) [23]. This product serves as the carbohydrate substrate for the synthesis of desired modified nucleosides by nucleoside phosphorylases (NP), most commonly by purine nucleoside phosphorylase (PNP, EC 2.4.2.1).

Another noteworthy area of medical biotechnology where RK is relevant is the enzymatic production of C-nucleosides, enabling biosynthesis of both therapeutic mRNA with reduced immunogenicity and C-nucleoside antibiotics. A variety of biocatalysts are capable of forming a β-C glycoside bond, utilizing RK product ribose-5-phosphate as a carbohydrate donor (Figure 1). Pseudouridine-5-monophosphate C-glycosidase (YeiN, EC: 4.2.1.70) accepts uracil as the nucleobase, and its substrate specificity tolerates substitution in both the pyrimidine and carbohydrate moieties [40,41]. Reported studies demonstrate metabolical engineering of a pseudouridine producer strain of *E. coli* and cascade synthesis of pseudouridine-5-monophphate (ΨMP) and its methylated analogue N^1^-methylpseudouridine-5-monophosphate [40,41,42,43]. One should also mention the C-glycoside synthases SdmA, OzmB and AlnA from the pathways of biosynthesis of the antibiotics showdomycin, oxazinomycin and alnumycin A respectively [44,45,46,47]. These enzymes also utilize ribose-5-phosphate (product of RK) to synthesize the C-nucleoside derivatives of pyrrole (SdmA), pyrazole (OzmB) and naphthoquinone (AlnA). To summarize, ribokinase can be utilized for the syntheisis of a broad variety of N- and C-nucleosides.

We should highlight that the described multienzymatic cascades for nucleoside and nucleotide synthesis can consist of biocatalysts from both mesophilic organisms and thermophiles such as bacteria from *Thermus* genus [30,31]. Performing the reactions at high temperature using thermostable enzymes offers several crucial advantages [48]. First, high temperature increases the solubility of nucleobases, which is often low in aqueous solution. Next, the recombinant enzyme formulation can contain contaminating enzymes from the producer strain, which can result in side reactions. Working at high temperatures allows deactivation of such mesophilic trace enzymes and prevents undesired product formation. High temperatures also decrease the solution’s viscosity and help to prevent bacterial/fungal contamination. In some cases, thermostable enzymes can be more resistant to proteolysis and chemical degradation. Moreover, the purification of thermostable enzymes can be simplified through thermal denaturation of satellite proteins.

Taking the advantages of thermostable enzymes into account, the development of thermostable ribokinases is a worthy challenge, especially since this is the starting biocatalyst not for one, but for two distinct cascades. We have previously reported two cascades of thermostable enzymes for the synthesis of nucleosides and nucleotides, both utilizing ribokinase from *Thermus* sp. 2.9 (*Tsp*RK) [30,31]. Unfortunately, *Tsp*RK is characterized by significantly lower activity compared to its mesophilic counterpart—ribokinase from *E. coli* (*Eco*RK) [30]. In this article, we report the design of the *Tsp*RK active site in order to increase its enzymatic activity for the cascade synthesis of modified ribonucleosides and ribonucleotides.

## 2. Results

### 2.1. Structure and Sequence Analysis

**Figure 2:**
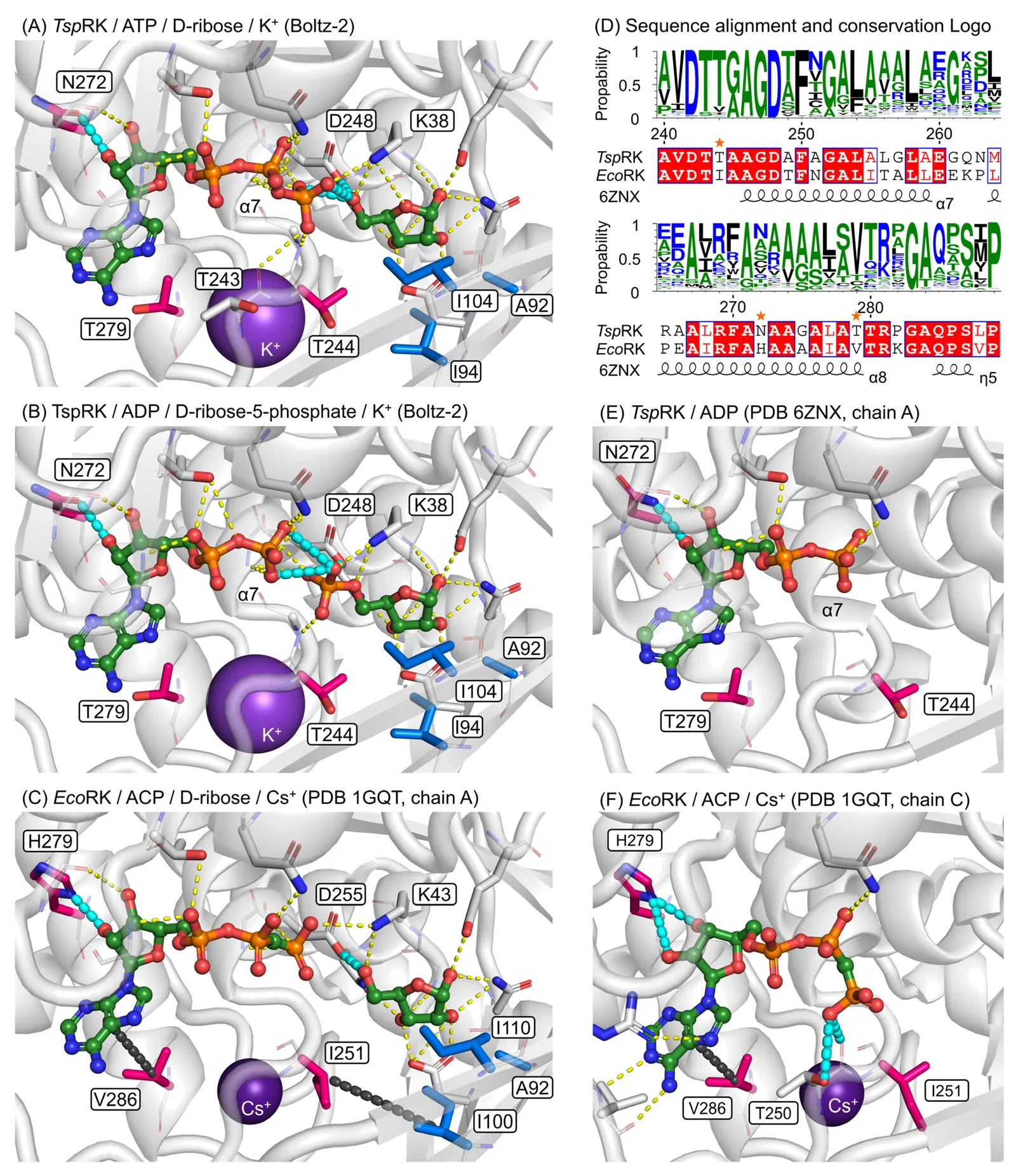
Boltz-2 models and X-ray structures of *Tsp*RK and *Eco*RK in complex with ligands, along with the sequence alignment and sequence conservation analysis. Residues selected for further investigation are highlighted in pink. Hydrophobic residues of the lid domain in *Eco*RK investigated in a previous paper (and their homologues in *Tsp*RK) are highlighted in blue [36]. In figure section F, interractions with D-ribose are not shown. Yellow dashes represent hydrogen bonds; cyan dashes highlight the most important interactions for the discussion of the catalytic mechanism. Dark gray dashes highlight hydrophobic interactions. In sequence logo, the residues are colored according to their properties: hydrophobic (black), neutral (green) and hydrophilic (blue). In sequence alignment, color higlights similarity, orange stars mark residues selected for further investigation. Below sequence aligment the symbols demonstrate the α-helixes in *Tsp*RK X-ray structure (PDB 6ZNX).

The optimization of the thermostable enzyme cascade necessitates the development of a biocatalyst that introduces pentose substrate into the synthesis pathway with maximum efficiency. We have determined that the wild-type *Tsp*RK has significant space for optimization in comparison to its mesophilic counterpart—ribokinase from *E. coli*. The latter enzyme has 10-fold larger specific enzyme activity under the desired reaction conditions. Thus, we have decided to engineer mutations in the active site of *Tsp*RK in order to improve its enzymatic activity. For this purpose, we have applied the consensus mutagenesis approach and compared *Tsp*RK and *Eco*RK for dissimilar active site residues that could be responsible for the difference in enzymatic activity.We have generated the models of *Tsp*RK in complex with its substates, products and ion cofactors with the Boltz-2 model and compared them with the existing X-ray structures of *Eco*RK and *Tsp*RK. Furthermore, we have performed sequence conservation analysis among ribokinase family enzymes. The resulting structure comparison, sequence analysis and conservation logo are presented in Figure 2.

Using Boltz-2, we have generated two types of models of *Tsp*RK in complex with its ligands: with the substrates ATP and D-ribose, along with the products ADP and D-ribose-5-phosphate (Figure 2A,B). Our model also includes the potassium ion cofactor. During attempts to include other ions such as Mg^2+^ and PO_4_^3−^, the position of these ligands in the model was inconsistent between subunits and dissimilar from the one observed in reported X-ray structures. Therefore, we have decided to limit our models to ligands whose predicted positions resemble reported experimental data to avoid artifacts.

Sequence alignment has shown 42% identity between *Tsp*RK and *Eco*RK. For comparative analysis, we used the X-ray structure of *Eco*RK (Figure 2C, PDB 1GQT) in complex with D-ribose and a non-hydrolyzable ATP analog, the compound commonly referred to as ACP (phosphomethylphosphonic acid adenylate ester). Moreover, we have compared the Boltz-2 model to the X-ray structure of *Tsp*RK in complex with ADP reported by us previously (Figure 2E, PDB 6ZNX) [49]. As we can see in Figure 2A,C, the positions of all ligands (substrates and potassium ion) are similar in both the *Tsp*RK Boltz-2 model and the *Eco*RK X-ray structure. The binding of ribose occurs through a network of hydrogen bonds. The same holds true for the model with the products and the X-ray structure of the *Tsp*RK/ADP complex (Figure 2B,E). Thus, this comparison validates the structures generated by Boltz-2.

The 5-hydroxyl group of ribose forms hydrogen bonds with the γ-phosphate of ATP and Asp248 (Asp255 in *Eco*RK). This residue is believed to play a catalytic role, being responsible for deprotonating the reactive hydroxyl group before the attack on the γ-phosphate of ATP [50]. Both in the ATP substrate and ribose-5-phosphate product, the transferred phosphate group is bound by the N-terminal fragment of α8-helix and residue Lys38. The remaining ATP molecule is bound by fewer polar contacts compared to the carbohydrate substrate.

**Figure 3:**
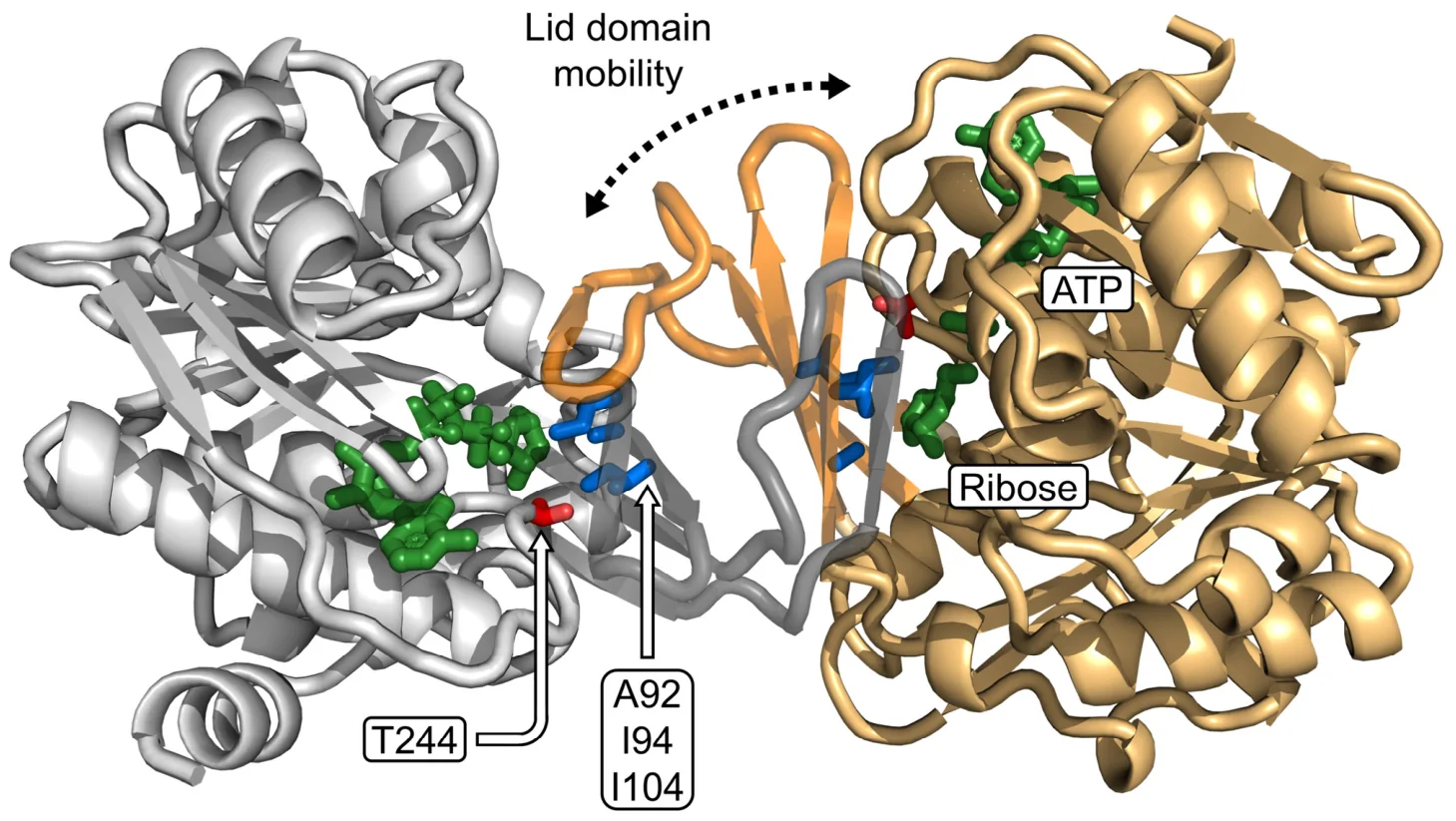
The tertiary structure of *Tsp*RK in the closed state in complex with ribose and ATP (Boltz-2 model). The two subunits of the homodimer are shown in grey and orange. The lid domains of the subunits (residues 9–36 and 91–107) are shown as semi-transparent and in a different shade of color compared to the remaining core α + β fold. The substrates are shown in green, residue Thr244 in red, and residues Ala92/Ile94/Ile104 in blue.

Further comparison of active sites of *Tsp*RK and *Eco*RK allows us to discover some key dissimilarities and formulate the hypothesis behind the difference in enzyme activity. The first point of interest is Thr244 in *Tsp*RK, homologous to Ile251 in *Eco*RK (Figure 2A–D). This residue is exposed to a triad of hydrophobic residues, namely Ala92, I94 and Ile104. In a previous article, we have shown that homologues of these three residues (Ala98, Ile100 and Ile110) play a key role in *Eco*RK activity and specificity towards carbohydrate substrates [36]. This cluster of hydrophobic residues is located on the so-called lid domain of the enzyme (Figure 3). During the transition towards the closed conformation, this domain covers the active site (hence the name), exposing the Ala/Ile residues towards the aliphatic hydrogen atoms of the ribose substrate [36,50,51,52].

As we can observe in Figure 2A,C and Figure 3, residues Thr244/Ile251 are located at the interface of interaction between the lid domain and the core α + β fold through the trio of hydrophobic residues of the former. Thus, we could speculate that the hydrophobic interactions between Ile251 and Ala98, and Ile100 and Ile110 in *Eco*RK play a positive role in catalysis, facilitating transition to closed conformation and supporting hydrophobic interaction with the carbohydrate substrate. Similar interactions through a moderately polar Thr244 in *Tsp*RK could be weaker, hypothetically contributing to the difference in enzymatic activity. However, we should acknowledge that Thr residues are the most common in the mentioned positions, followed by hydrophobic Val and Ile according to our sequence conservation analysis (Figure 2D). Nevertheless, excessive stabilization of the closed conformation could be harmful for catalysis. Thus, we hypothesize that the residues in Thr244 could play a role in modulating enzyme activity.

There are several other factors that make Thr244 an interesting research target. It is located in the immediate proximity to the area of phosphate transfer, separated by one residue from the α7-helix and three from catalytic Asp248. It should also be noted that Thr244 is located in the loop forming the pocket for monovalent cation cofactors, which are important for the ribokinase mechanism [53]. Moreover, the X-ray structure of *Eco*RK has an alternative conformation for the ATP mimetic ligand (Figure 2F). There, it interacts with Thr250—an immediate neighbor of the discussed Ile251 homolog of Thr244. Thus, we speculate that the residues in the position of Thr244/Ile251 could also affect catalytic activity and ATP binding.

The next target of interest that we have discovered is the Asn272 residue, which interacts with the 2′-hydroxyl group of the ATP substrate. In *Eco*RK, a similar interaction occurs through the His279 residue. A positive charge from the His residue could hypothetically have a positive effect on the pocket’s affinity towards a molecule with a high negative charge such as ATP. Curiously, our analysis shows that the residues in the discussed position are not conserved (Figure 2D). The second most frequent residue after Asn is Ala, which cannot form hydrogen bonds with its side chain. Ribokinase from *Arabidopsis thaliana* (UniProt A1A6H3) can serve as an example of an experimentally characterized enzyme that has an Ala residue in the discussed position [37]. Thus, our analysis suggests that the effect of Asn272/His279 is uncertain, but feasible for further research.

Finally, the third major difference that we have discovered is the Thr279 in *Tsp*RK in contrast to Val286 in *Eco*RK (Figure 2A,C). In the active site of *Eco*RK, the adenine ring of ATP is placed above the Val residues, suggesting a possible aliphatic-π hydrophobic interaction. Such interaction could be significantly weaker with a moderately polar, similarly shaped Thr residue in the *Tsp*RK active site. Here, the sequence conservation analysis supports our hypothesis, with Val being by far the most frequent variant in the discussed position (Figure 2D).

### 2.2. Affinity Prediction with Boltz-2

**Figure 4:**
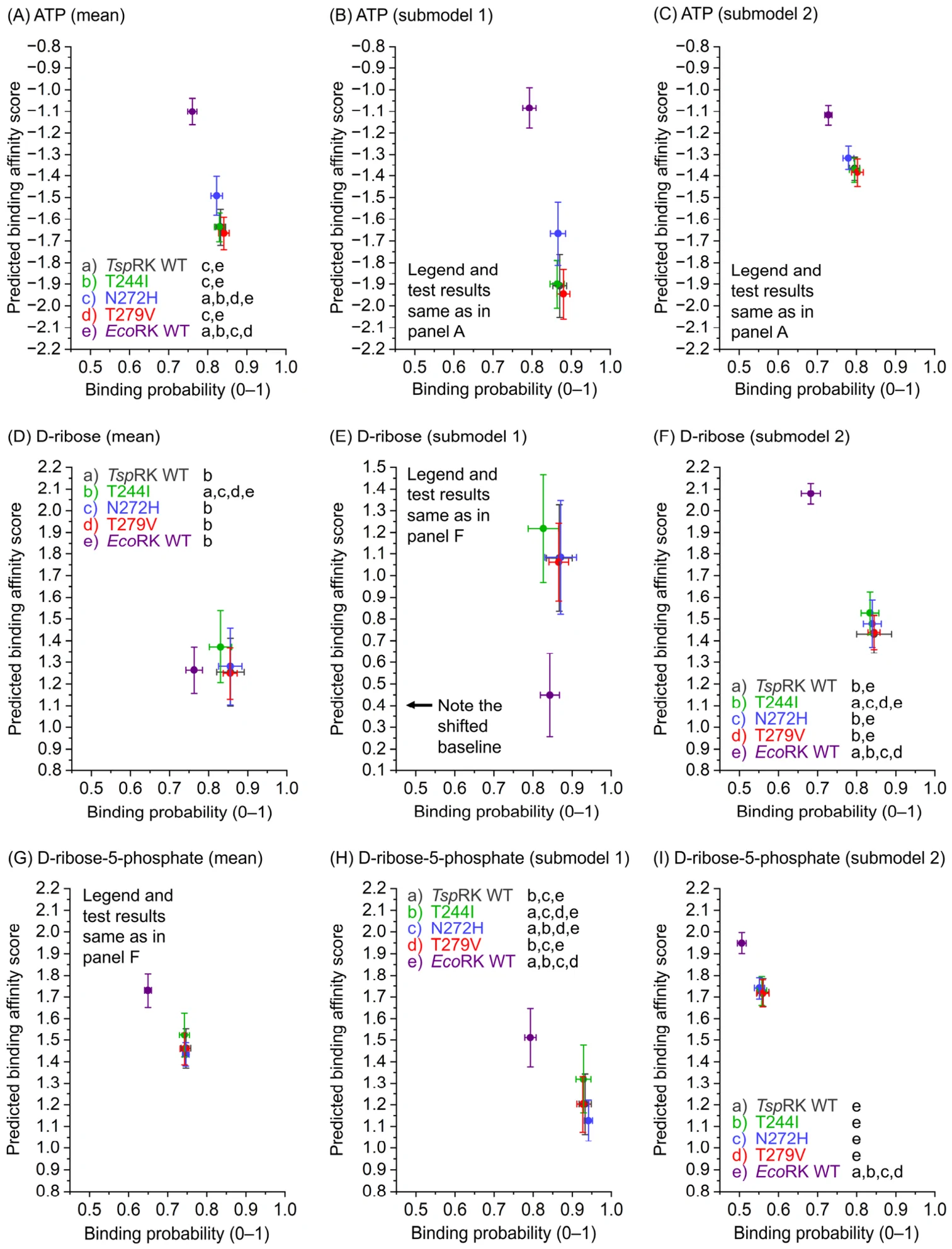
Results of Boltz-2 affinity prediction of *Tsp*RK and *Eco*RK towards ATP (**A**–**C**), D-ribose (**D**–**F**) and D-ribose-5-phosphate (**G**–**I**): predicted affinity and binding probability values (mean ± SD). Colors correspond to different enzymes: *Tsp*RK WT (dark grey), T244I (green), N272H (blue), T279V (red), and *Eco*RK WT (purple). Lowercase letters next to each variant in the legend denote pairwise statistical significance; a listed letter indicates a significant difference (*p* < 0.05) from that specific variant group based on a Kruskal-Wallis test with post-hoc Dunn’s correction (for example, «c,e» denotes a significant difference from variants c and e). All plots have the same scale. Panels representing data for ATP (panels (**A**–**C**)) have a different baseline for the predicted affinity values axis. Note that the axis for predicted affinity values is also shifted in panel (**E**).

Thus, our comparative analysis of *Tsp*RK and *Eco*RK active sites has allowed us to speculate that the mutations T244I, N272H and T279V could have a positive effect on the enzyme’s activity. In order to further assess their feasibility in silico, we have employed the Boltz-2 affinity prediction module. Each predicted model included two subunits of the *Tsp*RK dimer, a single copy of the investigated substrate, along with two copies of the complementary substrate and a potassium ion cofactor. In addition to the pair of substrates (D-ribose + ATP), we have performed affinity prediction for the product ribose-5-phosphate (modeled along with a pair of ADP molecules). Moreover, we have performed the estimations not only for wild-type and mutant *Tsp*RK, but also for *Eco*RK for reference. For each mutant–ligand pair, we have performed 100 independent runs. The statistical differences between predicted affinity values were tested with the Kruskal–Wallis test with the Bonferroni correction method, followed by Dunn’s test. The obtained results for the mean values between the two submodels are presented in Figure 4.

All of the predictions yielded high binding probability values (above 0.75), except for the results for ribose-5-phosphate from submodel 2. This result demonstrates that the mutations did not fundamentally disrupt the substrate binding mode in the active site of the enzyme. While comparing the predicted binding score between wild-type *Tsp*RK and the mutants, we observed differences that are within standard derivation (but are statistically significant). We could observe an increase in the score due to the N272H mutation for ATP and the T244I mutation for D-ribose and D-ribose-5-phosphate, along with a decrease in the score for D-ribose-5-phosphate due to the N272H mutation. Meanwhile, compared to the thermostable enzymes, the wild-type *Eco*RK displayed lower binding probability scores, along with elevated binding affinity scores for ATP and D-ribose-5-phoshoate. Moreover, we could observe a significant difference in predicted affinity values between submodels for the results for *Eco*RK with D-ribose. To summarize, the obtained results suggest that the selected mutations do not disrupt the substrate binding mode, but also do not strengthen the enzymes’ interaction with the investigated ligands. It should be noted that stronger substrate binding does not inherently mean improved catalysis.

To conclude, we employed Boltz-2 modeling, active site comparison and sequence conservation analysis to select mutations that could improve *Tsp*RK activity and are therefore feasible for experimental investigation: T244I, N272H and T279V.

### 2.3. Characterization of Enzymatic Activity

After the rational design of the active site, we performed site-directed mutagenesis and obtained the mutant forms of *Tsp*RK (along with the wild-type enzyme). Following the results of the investigation (described below), we combined the beneficial mutations in a double mutant enzyme, which was obtained and investigated as well. The producer strains (based on *E. coli* C3029/Tf16) were engineered and cultivated as described in the Materials and Methods Section. All enzymes were produced and purified in a similar manner. The recombinant enzymes constituted approximately 20% of the total cell protein (SDS-PAGE), and were partially in a soluble form that was subjected to further purification. We could not observe the mutations causing any significant change in recombinant enzyme production level, solubility or behavior during the purification process.

**Figure 5:**
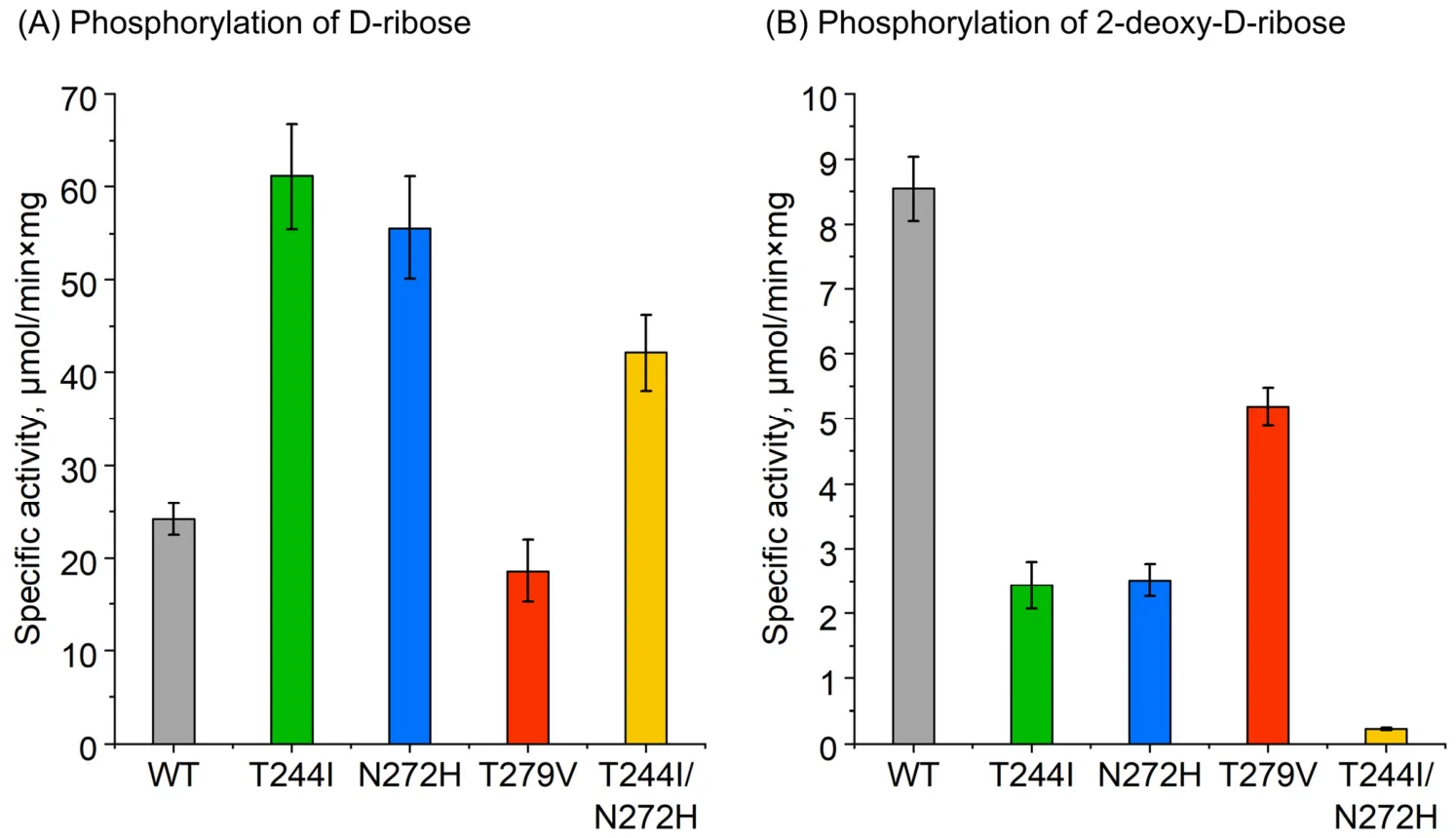
The specific activity of *Tsp*RK for D-ribose (**A**) and 2-deoxy-D-ribose (**B**) phosphorylation (mean ± SD): wild-type enzyme and mutant forms T244I, N272H, T279V and T244I/N272H. The concentrations of pentose and ATP substrates are 2 mM; the reactions were performed at 80 °C.

The activity of ribokinase was assayed through the determination of conversion of ATP to ADP via HPLC. The resulting enzyme-specific activity data are presented in Figure 5A.

As we can see in Figure 5, both T244I and N272H mutations have resulted in a 3-fold increase in specific activity. However, combining these two mutations has resulted in a slight decrease in specific activity relative to the single mutants. Meanwhile, we could not observe an increase in specific activity due to the T279V mutation.

We have also decided to determine the specific activity of the examined enzymes on unnatural carbohydrate substrates, using 2-deoxy-D-ribose as the example (Figure 5B). As a result, the mutations that benefited the natural reaction had a directly opposite effect on the unnatural substrates, with both T244I and N272H causing an approximately 4-fold decrease in activity compared to the wild-type enzyme, while the combined T244I/N272H has resulted in a further decrease by approximately 10 times compared to the single mutants. In contrast, the decrease in activity towards 2-deoxyribose due to the T279V mutation is more moderate (>2-fold). Thus, these results suggest that the observed positive effects of engineered mutations do not mean a similar effect can be observed for the unnatural substrates.

Another important factor for the application of engineered enzymes is their stability after prolonged incubation at elevated temperatures. We have compared the D-ribose phosphorylation activity of *Tsp*RK with and without a 2 h long pre-incubation at 80 °C in reaction buffer conditions without the substrates. As a result, both the wild-type enzyme and the T244I, N272H and T244I/N272H mutants have retained 70% of their specific activity. It should be noted that generally the presence of substrates is one of the positive factors for enzyme stability, and the examined mutant and wild-type *Tsp*RK maintain high activity even after incubation without substrates [54,55,56]. To summarize, the introduced mutations did not have a significant negative effect on enzyme stability in these experimental conditions.

Having identified that mutations T244I and N272H are beneficial for the natural reaction, we have proceeded to investigate their effects on the enzyme’s kinetic parameters. The calculated kinetic parameter results and Michaelis–Menten plots are reported in Table 1 and Figure S1.

During the investigation of kinetic parameters for D-ribose, we could observe a decrease in enzyme activity at high substrate concentrations. Therefore, the data for D-ribose were processed using the equation of substrate inhibition kinetics. In Table 1, we can observe that the *Tsp*RK affinity towards D-ribose is significantly higher compared to the second substrate ATP. Mutations T244I and N272H have increased *k_cat_* values by 3-fold and 2-fold respectively. Moreover, N272H has resulted in a decrease in *K_M_* (≈40%) and a nearly 3-fold increase in *K_i_*. Meanwhile, both mutations have increased the enzyme’s affinity towards ATP: We could observe a 4-fold reduction in *K_M_* for T244I and 2-fold reduction for N272H. To conclude, we successfully achieved the goal of our enzyme design project and engineered two mutant forms of ribokinase from *Thermus* sp. 2.9 with increased activity.

## 3. Discussion

Cascades of nucleic exchange enzymes are a highly feasible alternative to chemical synthesis of pharmaceutical nucleosides and nucleotides. Despite the advantages of highly specific reactions with no intermediate product purification, recombinant biocatalysts have their own limitations. Most importantly, in some cases, high consumption of recombinant enzyme can be a critical aspect for the technological feasibility of cascade synthesis. Nevertheless, recent developments in enzyme–ligand interaction modeling and rational active-site design allow us to overcome the limitations of natural biocatalysts.

We have successfully engineered two mutant forms of *Tsp*RK with a 3-fold increase in *k_cat_*. This result can be used to reduce the consumption of the biocatalyst during cascade synthesis. Ribokinase allows the utilization of the commodity reagent D-ribose as the donor of the carbohydrate moiety of both modified nucleosides and nucleotides. Thus, our result is relevant for optimization of two distinct cascade synthesis pathways: nucleoside synthesis via nucleoside phosphorylases and nucleotide synthesis via phosphoribosyltransferases.

The high thermal stability of *Tsp*RK makes our results especially relevant for the ribosylation of highly insoluble modified nucleobases. Moreover, in a previous research piece we have reported engineering a highly efficient biocatalyst for the synthesis of a promising class of antiviral compounds—phosphoribosylated derivatives of pyrazine-2-carboxamide [57]. Antiviral pyrazine-2-carboxamide derivative prodrugs such as compounds T-1105 (3-hydroxypyrazine-2-carboxamide) and T-705 (6-fluoro-3-hydroxypyrazine-2-carboxamide, known under the trademark «Favipiravir») undergo metabolic transformation into the active form in vivo by human HGPRT [58,59,60]. The efficiency of this process in human cells is very low, making the synthesis of phosphoribosylated pyrazine-2-carboxamide derivatives in vitro a viable direction for drug development [61,62,63]. We have previously engineered a mutant form of HGPRT from *Thermus thermophilus* with a >100-fold increase in the rate of synthesis of T-705 and T-1105 phosphorisbosides [57]. Constructing a cascade of multiple mutant thermostable enzymes with the newly designed *Tsp*RK mutants is one of the most immediate plans for future research.

Notably, a ribokinase from another thermostable organism, *Thermotoga maritima* (*Tma*RK), has been reported. Winkler has investigated 13 thermostable sugar kinases from this bacterium (including *Tma*RK) and reports applying them for the cascade synthesis of nucleotide triphosphates [64]. The reported cascades begin with nucleosides and nucleotides as starting sources, but theoretically could be extended to sugar pentoses using ribokinases such as *Tma*RK or the object of our research, *Tsp*RK. An earlier paper by Rodionova has measured the *Tma*RK activity to be 8.5 μmol/min×mg [65]. This value is twice as low as the value reported by us for the wild-type *Tsp*RK. Nevertheless, for reliable comparison, the two enzymes should be obtained and assayed in parallel under identical conditions, including characterization of kinetic parameters. Furthermore, it would be interesting to compare these enzymes in terms of their thermal stability under prolonged incubation in reaction conditions and substrate inhibition properties. Finally, another important question: what effect would the mutations performed by us on *Tsp*RK have on *Tma*RK activity?

It is also worth acknowledging the limitations of the engineered biocatalysts and areas for further improvement. First, the positive effects of T244I and N272H mutations are relevant for the natural reaction and cannot be automatically extrapolated to other non-natural pentoses. While using 2-deoxy-D-ribose as the substrate, the T244I and N272H mutations had a directly opposite effect—a significant reduction in specific activity for the unnatural substrate. This difference between the natural and unnatural reactions can be hypothetically attributed to the way these mutations affect ATP binding. Both T244I and N272H substitutions have resulted in an increase in the enzyme’s affinity towards ATP through multifold lower *K_M_* values. We speculate that these mutations could favor the stabilization of the ATP position in the active site, thus positively contributing to the natural reaction. However, the conformation of ATP that is more favorable for 2-deoxyribose phosphorylation could be slightly different from the one that is optimal for the natural reaction. If this hypothesis is true, then stabilization of the ATP conformation would be favorable for the natural reaction but would negatively affect the phosphorylation of unnatural pentoses. Further research is required to determine the reasons behind the observed effect.

For both the wild-type and mutant enzymes, we could observe inhibition by the natural substrate D-ribose at elevated concentrations. We should note that frequently the limiting factor for the working substrate concentrations is the solubility of the nucleobase. One of the intended purposes of the development of thermostable nucleic exchange enzymes is to increase the working temperature while working with poorly soluble nucleobases. For this application, nucleobase solubility is the factor limiting the working concentration of the substrates. Nevertheless, if the target nucleobase is relatively highly soluble and allows high substrate concentrations, the substrate inhibition observed in both wild-type and mutant enzymes would be a challenge. In this case, it would be preferable to use ribokinases that do not display inhibition by D-ribose, such as *Eco*RK.

While discussing substrate inhibition, we should also note that N272H has caused both a slight decrease in *K_M_* and an increase in *K_i_* for the carbohydrate substrate D-ribose. The target residue of this mutation, Asn272, is responsible for ATP binding, so one could expect that the N272H effect on ribose binding to the enzyme is complex and indirect. There are multiple factors that could affect the inhibition of *Tsp*RK by its natural carbohydrate substrate, such as open–closed state transition dynamics, interaction between ATP and ribose during substrate binding in correct/incorrect pose, allosteric effects and others. Explanation of the observed effect requires further research on the precise mechanism of inhibition by both the wild-type and mutant enzymes.

Next, it is important to discuss an issue that is critical for the future application of the engineered biocatalyst: enzyme stability. There was a possibility that the engineered mutations would significantly decrease enzyme stability during prolonged incubation at high temperatures. We have tested and shown that both the wild-type and mutant enzymes retained 70% of their activity after 2 h of incubation at 80 °C. The experimental reaction mix did not contain the substrates, because otherwise the enzymatic reaction would begin. The observed 30% reduction in activity could be caused by the absence of substrates that could play a role in stabilizing the enzyme [54,55,56]. Thus, the engineered mutations did not cause a large drop in enzyme stability from our initial assessment. Ribokinase may be applied for a variety of enzyme cascades with different reaction conditions, so further research is necessary to determine how the engineered mutants will function in precise applications.

Another aspect of our results that is worthy of discussion is the application of the Boltz-2 model for rational design. The affinity prediction module results suggested that, on one hand, the introduced mutations do not disrupt the binding modes that are favorable for catalysis. On the other hand, the investigated substitutions do not substantially enhance binding affinities, despite the experimentally observed multifold decrease in *K_M_* towards ATP by both mutants. It must be highlighted that catalysis is a complex process which cannot be limited to the strength of substrate binding alone. The probable factors behind the positive effects of the T244I mutation are especially complex, including diverse hypothetical mechanisms. The position of Thr244 suggests that it could affect interactions with the lid domain, open–closed enzyme conformation transition, ATP binding and interaction with monovalent cation cofactors. We should also note that our model does not include the Mg^2+^ and PO_4_^3−^ ions as a potential source of artifacts: Boltz-2 predicted positions that were inconsistent between the model’s two subunits and did not resemble existing experimental data. Nevertheless, the influence of these ions on catalysis is an important factor that must be taken into account in further research. To summarize, the presented results and factors discussed above highlight how enzymatic catalysis is a multi-factor process that cannot be limited to the active site affinity towards the substates. Further research is necessary for a conclusive interpretation of the obtained results.

## 4. Materials and Methods

### 4.1. Molecular Modeling and Sequence Conservation Analysis

Structural visualization, analysis, and editing were conducted in PyMOL 2.5. Multiple sequence alignment was carried out using MAFFT 7.490, followed by rendering with ESPript 3.0 and WebLogo 3.3 [66,67,68]. Ribokinase sequences for conservation analysis were selected from the UniProt database annotated as EC 2.7.1.15 with gene name “rbsk”, “rbks” or “rbk” and sourced from reference proteomes (keyword KW-1185). A total of 14,330 sequences were subjected to further analysis.

For Boltz-2 (version 2.10.8) structure and affinity prediction, sequences of *Tsp*RK and *Eco*RK were subjected to multiple sequence alignment using the MMseqs2-based web service provided by ColabFold and accessed through Boltz-2’s “--use_msa_server” option [69,70,71]. Each predicted system included two enzyme subunits, a single copy of the investigated ligand molecule, two copies of the complementary second substrate/product and two copies of the potassium ion cofactor. The ligands were defined through SMILES sequences provided in Supplementary Table S1 (along with a sample of an input .yaml file). For each ligand/enzyme system, we performed 100 independent runs of structure and affinity prediction. The statistical significance of differences in obtained predicted affinity values (*p* < 0.05) was determined via Kruskal–Wallis test with post hoc Dunn’s test using Statistics Kingdom online tool (https://www.statskingdom.com/kruskal-wallis-calculator.html accessed on 03 July 2026).

### 4.2. Producer Strain Engineering and Cultivation

All chemicals used in the research were of laboratory grade and obtained from commercial suppliers and were used without further purification. The recombinant *Tsp*RK producer strains were constructed based on C3029/Tf16 strain *E. coli* cells (C3029 New England Biolabs, Ipswich, MA, USA; pTf16 plasmid Takara Bio, Kusatsu, Japan). The strain cells were transformed with a plasmid containing the gene encoding wild-type ribokinase from *Thermus* sp. 2.9 (GenBank: KHG65688.1) with a C-terminal His-tag used in a previous study, or the mutant enzyme genes obtained through site-directed mutagenesis [30].

All of the recombinant enzymes (wild-type and mutants) were produced and purified through an identical scheme. The cultures of the producer strains were cultivated in liquid LB medium (per liter: 10 g tryptone, 5 g yeast extract, 10 g NaCl, supplemented with 100 μg/mL ampicillin, 20 μg/mL chloramphenicol, 1 mg/mL L-arabinose). The cells were cultivated at 37 °C until reaching an optical density of OD_600_ = 0.8, followed by cooling and supplementation with 0.4 mM IPTG. After induction, the producer strains were cultured for 14 h at 23 °C and harvested by centrifugation. The recombinant *Tsp*RK was obtained partially in soluble form.

### 4.3. Enzyme Isolation and Purification

The wet cell biomass was dissolved (1:10 *w*/*v*) in a buffer with 50 mM Tris-HCl pH 8.0, 1 mM benzamidine hydrochloride, and 1 mM PMSF. The resuspended cell biomass was subjected to ultrasonic disintegration and centrifugation at 4000× *g*. The supernatant was heated for 15 min at 65 °C; the denatured satellite proteins were removed with a second round of centrifugation.

The resulting supernatant was applied onto an XK16/20 column, packed with 20 mL of Protino Ni^2+^-IDA sorbent (Macherey-Nagel, Düren, Germany), which was pre-equilibrated with Buffer A (50 mM Tris-HCl pH 8.0). The column was washed with 5 CV of Buffer A, followed by 5 CV of Buffer B (50 mM imidazole, 50 mM Tris-HCl pH 8.0). Recombinant *Tsp*RK was eluted with 5 CV of Buffer C (200 mM imidazole, 50 mM Tris-HCl pH 8.0).

Fractions containing *Tsp*RK according to SDS-PAGE analysis were pooled and concentrated on the Ultracel 30 kDa membrane (PLTK06210, Millipore, Burlington, MA, USA). The resulting concentrate was applied onto the HiLoad 16/60 column packed with Superdex 75 sorbent (GE Healthcare (Cytiva, Marlborough, MA, USA), which was previously equilibrated with Buffer E (50 mM Tris-HCl pH 7.4, 5 mM MgCl_2_, 5% glycerol, 0.04% NaN_3_). During gel filtration, the target enzyme was eluted with Buffer E; fractions containing *Tsp*RK were pooled and concentrated on the Ultracel 30 kDa membrane to a concentration of approximately 10 mg/mL. Protein purity and concentration were assessed using SDS-PAGE and the Bradford assay, respectively [72,73]. The resulting solutions of recombinant *Tsp*RK (wild-type and mutant forms) were aliquoted and stored at −80 °C for further analysis.

### 4.4. Investigation of Enzymatic Activity and Kinetic Parameters

The reaction mix (100 µL) for the determination of enzymatic activity consisted of: 2 mM D-ribose/2-deoxyribose, 2 mM ATP, 50 mM Tris-HCl pH8, 50 mM KCl, 5 mM MgCl_2_, 1 mM KH_2_PO_4_. Reaction mix for the determination of kinetic parameters towards D-ribose consisted of 0.05–6 mM D-ribose, 2 mM ATP, 50 mM Tris-HCl pH8, 50 mM KCl, 5 mM MgCl_2_, 1 mM KH_2_PO_4_. Reaction mix for the determination of kinetic parameters towards ATP consisted of 0.1-6 mM ATP, 2 mM D-ribose, 50 mM Tris-HCl pH8, 50 mM KCl, 5 mM MgCl_2_, 1 mM KH_2_PO_4_. The samples were preheated before adding 0.2 μg recombinant *Tsp*RK, followed by 4 min of incubation at 80 °C. During the determination of the enzyme stability experiment, the enzyme samples were pre-incubated in 50 mM Tris-HCl pH 8, 50 mM KCl, 5 mM MgCl_2_, 1 mM KH_2_PO_4_ for 2 h (with the control being stored at 4 °C for the same time). After pre-heating, the enzyme sample solution was added to pre-heated aliquots of the reaction mixes, thus initiating the substrate conversion. All of the reactions were stopped by diluting the sample with >10-fold volume of ice-cold buffer containing 100 mM Na_2_SO_4_, 50 mM TRIS-HCl pH 7.2. The assay was performed in 3 replicates.

Ribokinase activity was determined by measuring the conversion of the substrate ATP into the product ADP via HPLC, performed on the Hitachi Chromaster system (Pump 5110, Column oven 5310, UV Detector 5410, Chiyoda, Tokyo, Japan). Analysis was performed on the Supelcosil LC-18-T column, 5 µm, 150 × 4.6 mm (Supelco/Sigma-Aldrich, Bellefonte, PA, USA), with the following method: isocratic elution with 100 mM KH_2_PO_4_, 50 mM TEAA (tetraethylamine acetate) pH 5.3, flow rate 1 mL/min, detection at 254 nm, yielding clean separation of the target analytes. One unit of *Tsp*RK activity was defined as 1 µmol of ribose substrate converted into product per 1 min per 1 mg of recombinant enzyme. The kinetic parameters were determined through nonlinear regression analysis on the mean values of specific activity at varying concentrations using the SciDAVis v2.3.0 software. The catalytic constants were calculated for one enzyme subunit, assuming a molecular weight of 32.0 kDa according to the amino acid sequence. The kinetic parameters were determined using the Michaelis–Menten equation for ATP data and the substrate inhibition equation for D-ribose data:

(1) $$V=\frac{V_{max}\;\times S\;}{K_{M}\;+\;S\;\times (1\;+\;\frac{S}{Ki})}$$

## Figures and Tables

**Table 1:** Kinetic parameters of the wild-type *Tsp*RK and the T244I and N272H mutants (along with data for *Eco*RK from a previous study) [36].

	*V_max_*, μmol/min × mg	*k_cat_*, s^−1^	*K_M_*, mM	*k_cat_*/*K_M_* M^−1^ × s^−1^	*K_i_*, mM
**D-ribose**					
*Tsp*RK WT	32 ± 3	17 ± 2	0.23 ± 0.02	7.4 × 10^4^	1.8 ± 0.1
T244I	92 ± 7	49 ± 4	0.40 ± 0.03	1.2 × 10^5^	2.0 ± 0.2
N272H	71 ± 2	38 ± 1	0.14 ± 0.01	2.7 × 10^5^	5.6 ± 0.2
*Eco*RK WT [36]	340 ± 50	180 ± 30	0.15 ± 0.02	1.2 × 10^6^	-
**ATP**					
*Tsp*RK WT	38 ± 4	20 ± 2	2.7 ± 0.3	7.4 × 10^3^	-
T244I	60 ± 2	32 ± 1	0.67 ± 0.02	4.8 × 10^4^	-
N272H	88 ± 3	47 ± 2	1.18 ± 0.04	4.0 × 10^4^	-
*Eco*RK WT [36]	350 ± 40	185 ± 21	0.43 ± 0.05	4.3 × 10^5^	-

## Data Availability

The data presented in this study are available on request from the corresponding authors.

## Supplementary Materials

The following supporting information can be downloaded at: https://www.mdpi.com/article/10.3390/ijms27198911/s1.

## Abbreviations

The following abbreviations are used in this manuscript:

ACP	Phosphomethylphosphonic acid adenylate ester
AlnA	Alnumycin A pathway C-glycoside synthase
APRT	Adenine phosphoribosyltransferase
CV	Column volume
HGPRT	Hypoxanthine-guanine phosphoribosyltransferase
NAMPRT	Nicotinamide phosphoribosyltransferase
NP	Nucleoside phosphorylase
OzmB	Oxazinomycin pathway C-glycoside synthase
PNP	Purine nucleoside phosphorylase
PP_i_	Inorganic pyrophosphate
PPM	Phosphopentomutase
PRPP	Phosphoribosylpyrophosphate
PRPPs	Phosphoribosylpyrophosphate synthetase
PRT	Phosphoribosyltransferase
RK	Ribokinase
SdmA	Showdomycin pathway C-glycoside synthase
YeiN	Pseudouridine-5-monophosphate C-glycosidase
