# Supplementary material for: Enhancing the Activity of Ribokinase from Thermus sp. 2.9 Through Rational Design for the Cascade Synthesis of Modified Nucleotides and Nucleosides
Source: Int J Mol Sci. 2026 Oct 7;27(19):8911. doi: 10.3390/ijms27198911 (PMC13665051; doi:10.3390/ijms27198911)
Supplement: Supplementary file 1 [file ijms-27-08911-s001.zip › ijms-4521773-supplementary.pdf]

Table S1: SMILES sequences used to define the ligands during Boltz-2 prediction and a sample of the .yaml input file

Ligand	SMILES
D-ribose	`OC[C@H]([C@H]([C@H]1O)O)O[C@@H]1O`
D-ribose-5-phosphate	`[O-]P([O-])(OC[C@H]([C@H]([C@H]1O)O)O[C@@H]1O)=O`
ATP	`Nc1c2nc[n]([C@@H]([C@@H]3O)O[C@H](COP([O-])(OP([O-])(OP([O-])([O-])=O)=O)=O)[C@H]3O)c2ncn1`
ADP	`Nc1ncnc2c1nc[n]2[C@@H]([C@@H]1O)O[C@H](COP([O-])(OP([O-])([O-])=O)=O)[C@H]1O`
Potassium ion	`[K+]`

Sample of the .yaml input file


```
version: 1
sequences:
  - protein:
      id: [A, B]
      sequence:
MILVVGSLNMDLVLRVKRLPRPGETVLGEDYQTHPGGKGANQAVAIARLGGKVRMLGRVGEDPFGQALKSGLAQEG
VDVAWVLETPGPSGTAFILVDPEGQNQIAVAPGANARLVPEDLPATAFQGVGVVLLQLEIPLETVVRAAALGRKAG
ARILLNAAPAHALPSEILQSVDLLLVNEVEAAQLTEASPPRTPEEALALARQLRGRAPQAQVVLTLGAQGAVWSGT
EESHFPAFPVRAVDTTAAGDAFAGALALGLAEGQNMRAALRFANAAGALATTRPGAQPSLPFRDEVEALLFG
      msa: .../NNN.csv
  - ligand:
      id: [C]
      smiles: "OC[C@H]([C@H]([C@H]1O)O)O[C@@H]1O"
  - ligand:
      id: [D, E]
      smiles: "[K+]"
  - ligand:
      id: [F, G]
      smiles: "Nc1c2nc[n]([C@@H]([C@@H]3O)O[C@H](COP([O-])(OP([O-])(OP([O-
])([O-])=O)=O)=O)[C@H]3O)c2ncn1"
properties:
  - affinity:
      binder: C
```

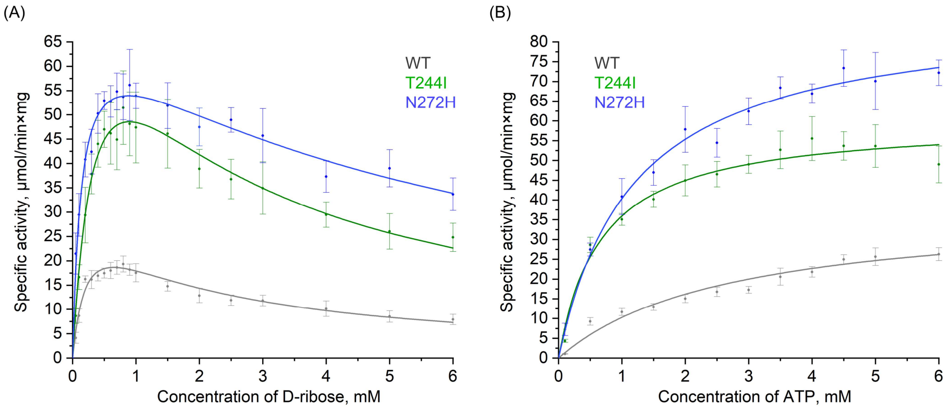

Figure S1: The Michaelis-Menten plots showing the fitting of the calculated curve (shown as a line) to the mean values of specific activity of *Tsp*RK (wild type and mutant forms T244I and N272H) at different concentrations of: A) D-ribose, B) ATP. The reactions were performed at 80°C.
